# Secondary analysis of the intestinal microbiota of healthy chickens

**DOI:** 10.1016/j.psj.2026.106915

**Published:** 2026-04-13

**Authors:** Matheus Barros, Andrea Pietruska, Zubair Khalid, Joseph P. Gulizia, Ruediger Hauck

**Affiliations:** a Department of Poultry Science, Auburn University, Auburn, AL 36849, United States; b Department of Pathobiology, Auburn University, Auburn, AL 36849, United States

**Keywords:** Microbiome, Gut, Poultry

## Abstract

The intestinal microbiota plays a key role in poultry health and performance. Its composition is influenced by multiple factors such as diet, age, bird type, and housing. The aim of this secondary analysis was to summarize existing data and outline a baseline reference intestinal microbiota that could serve as a reference for future research and industry applications. For this, the intestinal microbiota of chickens across all intestinal segments and feces under standard conditions, without feed additives or infections were compiled and analyzed. A total of 3,562 samples from 79 BioProjects were retrieved from the National Center for Biotechnology Information database, together with their metadata including intestinal segment, bird type, age, housing system, continent, and sequenced hypervariable region. Across all samples, 2,603 unique bacterial genera in 73 phyla were identified, with Firmicutes being the most abundant phylum in all segments. The number of core genera was highest in the ceca, followed by the duodenum. Alpha diversity was highest in the ceca and lowest in feces, as well as highest in Africa and lowest in North America. For beta diversity, Principal Component Analysis revealed a minor overlap among intestinal segments and a major overlap for the other investigated factors. Metabolic pathway predictions revealed 8,674 unique functional orthologs (FOs), with the relative abundance of about 40 to 60% of the FOs constant across intestinal segments and other investigated factors. In conclusion, while microbial community composition may vary substantially between different flocks or production systems, core metabolic functions are often consistent. These findings provide a baseline framework for evaluating how treatments, infections, or management practices may impact the chicken intestinal microbiota.

## Introduction

Understanding the structure and functionality of the intestinal microbiota, as well as the symbiotic interactions between the host and microorganisms, is fundamental for the health and production of poultry (Shang et al., 2018; Carrasco et al., 2019). To adequately assess variations in the microbiota of poultry populations, it is essential to establish a representative baseline of what would be considered in the range of a “normal” microbiota. However, the insufficient knowledge of bacterial diversity, both phylogenetic and functional, in poultry intestines has created a significant gap that hinders the understanding of these interactions (Wei et al., 2013; Yue et al., 2024).

Currently, the 16S ribosomal RNA (rRNA) gene sequencing technology is the predominant method for studying bacterial communities, including in the poultry intestine, due to its throughput, accuracy, and cost-effectiveness (Stanley et al., 2014; Cardenas et al., 2021). 16S rRNA gene sequencing has revealed a more complex and diverse microbiome than classical bacteriology based on culturing bacteria (Oakley et al., 2014), offering a comprehensive overview of the microbial community (Stanley et al., 2014). Currently, platforms like Illumina are the predominant choice used for 16S rRNA gene sequencing.

Several factors can influence the composition of poultry intestinal microbiota, such as age, breed, intestinal region, sex, diet, bedding type, medications, and diseases (Kers et al., 2018). These factors can lead to significant variations in the microbial communities present in the poultry gastrointestinal tract, affecting their functionality and interactions with the host. Understanding these variations is critical for developing strategies to modulate microbiota and enhance poultry health and production efficiency. However, the most significant limitation is the lack of available data and comprehensive metadata descriptions, which are crucial for evaluating these influences and their correlations (Cardenas et al., 2021).

In microbiome analysis, various key parameters provide a comprehensive understanding of the microbial community’s structure and functionality, such as taxonomy, core microbiota, alpha diversity, beta diversity, relative abundance, and predicted metabolic function. These parameters collectively evaluate the diversity within and between samples, the composition and relative abundance of microbial species, and their potential metabolic activities based on gene sequencing data. This holistic approach allows to assess the ecological and functional dynamics of the microbiome, offering insights into its impact on the host’s health and productivity (Caporaso et al., 2011; Langille et al., 2013).

Previous studies have provided critical insights into the role of intestinal microbiota in poultry health. Wei et al. (2013) conducted a bacterial census of the poultry intestinal microbiome, which has served as a reference for subsequent studies in this field. Similarly, Cardenas et al. (2021) performed a secondary analysis of the cecal microbiota of chickens, showing challenges in data availability, metadata description, and sources of variation such as primer choice and pathogen presence. However, none of these studies examined all intestinal segments or identified the relationships with the factors influencing the microbiota.

In this study, a secondary analysis was conducted to compile and analyze the intestinal microbiota of all intestinal segments and feces of chickens that were left untreated with feed additives, uninfected, and otherwise kept close to standard conditions. The purpose of this study was to summarize and analyze the data and to possibly define a baseline reference intestinal microbiota, which could serve as a reference for future research and industry practices.

## Materials and methods

### Data collection

The National Center for Biotechnology Information (NCBI) BioProjects database was searched on September 5th, 2023. The search terms used were: (chick* OR broiler OR layer OR hen) AND (micro* OR 16S OR Illumina) AND (intestine* OR gut OR duodenum OR jejunum OR ileum OR cecum). The following inclusion criteria were applied to the BioProjects: (1) They were linked to raw reads on the Sequence Read Archive (SRA). (2) 16S rRNA next generation amplicon sequencing was conducted on a Illumina platform. Experiments that used other next generation sequencing platforms were excluded to prevent biases arising with data obtained by different sequencing methods. (3) The linked reads were obtained from controlled experiments investigating the intestinal microbiota of chickens, i.e. results of field studies were excluded. (4) There was an associated article in a peer-reviewed journal describing the experimental design. (5) The experimental design contained a control group that was left uninfected and not treated with anti-, pre-, pro-, or synbiotics or phytogenic feed additives. (6) The necessary metadata was available for each sample of the selected BioProjects. If several groups met the previous definition, e.g. in nutritional studies, the group that was considered to best represent commercial practices was selected.

The following factors were extracted for each sample from the articles linked to the selected BioProjects or the metadata available on SRA papers: Intestinal segment (duodenum, jejunum, ileum, ceca, feces), bird type (white broiler, layer, other), days of age (doa), housing (cages, floor pens, change of housing type during experiment), continent, and sequenced hypervariable region. Data search and data extraction were completed independently by two reviewers (MS and RH), and discrepancies were decided in consensus after revisiting the metadata and/or associated publication.

### Preprocessing of sequences in QIIME 2

The code used for preprocessing of the raw sequences in QIIME 2 and all following steps, is available at: https://github.com/PoultryPathologyAuburn/Matheus_MicrobiomeMetaAnalysis

Raw data files in fastq format were imported and downloaded from SRA using the faster-dump function of the sratoolkit (Alvarez et al., 2017). Sequences of each study were imported separately into QIIME 2 (Bolyen et al., 2019) as either paired-end or single-end reads, depending on the data type. If one study included samples from more than one intestinal segment, the sequences were imported separately for each segment. This combination of study and intestinal segment will henceforth be referred to as “data set”.

Primers were trimmed and reads were truncated and denoised, merged, filtered for chimeras and finally assigned to amplicon sequencing variants (ASVs) using the dada2 plugin (Callahan et al., 2016). Cutting and truncating parameters were optimized for each data set to maximize the number of obtained reads (Multimedia Component 2). Representative sequences and the feature table were exported for analysis of inferred metabolic pathways.

Taxonomy tables were generated using the Silva 138 SSURef NR99 full-length sequences classifier (Quast et al., 2013; Robeson et al., 2021) to obtain taxonomic compositions of the samples from each experiment. The data were consolidated into a single taxonomy file and a sample table organized by intestinal segment, then exported.

### Preprocessing in R

Taxonomic composition, alpha diversity, and beta diversity were analyzed in R version 4.3.2 (R Core Team, 2023) and R studio version 2023.12.0 (Posit Team, 2024). Sample tables, taxonomies, and metadata for each data set were imported using the qiime2R and phyloseq packages for R (McMurdie and Holmes, 2013; Bisanz, 2025). After filtering phyloseq objects to retain only bacterial information, excluding mitochondria and chloroplasts, samples with less than 10,000 reads and taxa with zero counts were pruned. The dplyr package (Wickham et al., 2023) was used to extract descriptive data and for all subsequent data manipulation steps.

### Taxonomy

The number of unique genera and phyla in each data set and overall was extracted from the phyloseq objects corresponding to each intestinal segment. For the taxonomy bar plot and the extraction of core genera, the average relative abundance of each taxon in each data set was calculated. The taxonomy bar plot was generated using the mean of average relative abundances from each data set to avoid bias from differing sample numbers with the ggplot2 package (Wickham, 2016).

Genera were considered core if they were present in more than a certain percentage of samples within more than a certain percentage of data sets for a given intestinal segment. For both thresholds, i.e. the prevalence of a genus in each data set and the percentage of data sets in which its prevalence exceeded the first threshold, values of 50, 60, 70, 80 and 90% were tested. The number of core genera, the cumulative percentage of core genera of all genera detected in the intestinal segment, and cumulative average relative abundance of core genera were recorded for each combination of the two thresholds for each intestinal segment.

### Alpha diversity

Simpson’s diversity index for richness and Shannon diversity index for evenness were calculated for every sample. Normality was assessed by inspection of histograms, Shapiro-Wilk test and Levene’s. While normality could be assumed for Shannon diversity index, Simpson’s diversity index was rank normalized so that the following analysis reflected relative ordering of diversity rather than absolute differences. For both variables, a linear mixed-effects model was fitted using the lmer function of the lme4 package (Bates et al., 2015), with intestinal segment, continent, doa, bird type, housing, and hypervariable region as predictors and BioProject as a random effect. Predictors that yielded non-significant linear relationships with the response variable were removed from the model. All models were tested for collinearity of predictors using the vif function of the car package (Fox and Weisberg, 2019). Akaike Information Criterion (AIC) values were calculated for all models using the AIC functions of the Stats package (R Core Team, 2013). Values predicted by each model and actual values were visualized using the ggplot2 package.

### Beta diversity

For beta diversity evaluation, the merged phyloseq object containing all samples was collapsed to the genus level. Principal Coordinates Analysis (PCoA) plots were generated using the ordinate and plot_ordination functions of the phyloseq package to visually assess the beta diversity. For Permutational Multivariate Analysis of Variance (PERMANOVA), the distance matrix was calculated and ordinated based on Bray-Curtis dissimilarity. Because PERMANOVA can be sensitive to heterogeneity in group dispersion, homogeneity of dispersions was analyzed by the betadisper function followed by ANOVA and by the permutation test of the vegan package (Oksanen et al., 2025). PERMANOVA was conducted using the adonis2 function of the vegan package with 999 permutations. Intestinal segment, continent, doa, bird type, housing, and hypervariable region were tested as factors. Because PERMANOVA does not allow inclusion of continuous factors, age categories were tested. The categories were less than 5 doa, 5 to 9 doa, 10 to 19 doa, 20 to 49 doa, and older than 50 doa.

### Pathways and metabolic prediction

The analysis of inferred pathways was performed with PICRUSt2 version 2.5.1 (Douglas et al., 2020). The outputs were imported into R and combined into one data set. Data were manipulated and visualized using the dplyr, tidyr, and ggplot2 packages (Wickham et al., 2024).

Differential detection of functional orthologs (FOs) was analyzed using the DESeq2 package, based on the negative binomial distribution. The analysis was performed with the DESeq function, which includes estimation of size factors, estimation of dispersion, negative binomial general linear model (GLM) fitting Wald statistics (Love et al., 2014) and adjusting p-values with the Benjamini–Hochberg method. Count data were imported and normalized to account for differences in sequencing depth. Differentially abundant FOs (DFOs) were filtered out using statistical significance thresholds of Padjust < 0.05 leaving only non-differentially abundant FOs (nDFOs). Intersections of nDFOs identified in several analyses were visualized by upset plots created with the upset function using the UpSetR package (Conway et al., 2017). Metabolic pathways containing more nDFOs than expected by chance were identified using the enrichKEGG function of the clusterProfiler package (Xu et al., 2024) Raw p‑values for each pathway were adjusted for multiple comparisons using the Benjamini–Hochberg method.

## Results

### Description of BioProjects

The initial literature search resulted in 640 BioProjects, of which 85 met all inclusion criteria (Multimedia Component 1). However, 4 BioProjects had no valid fastq files available and fastq files of 2 samples from 2 different BioProjects could not be downloaded, resulting in 4,352 samples from 81 BioProjects (Multimedia Component 3). Total samples were filtered during pre-processing to include only bacterial sequences and samples with more than 10,000 remaining reads, and as a result, 3,562 samples from 79 BioProjects were retained (Multimedia Component 3). Five, 18, 24, 63, and 9 BioProjects investigated microbial communities from the duodenum, jejunum, ileum, ceca, and feces, respectively (Table 1). Thirty-six BioProjects were from Asia, 25 from Europe, 13 from North America, 2 from Africa, and 3 from Hawaii. Fifty-five BioProjects used commercial broilers, 13 commercial layers, and 14 other breeds, including yellow broilers and indigenous breeds. Bird type was not specified in one BioProject. Birds were housed in cages in 35 BioProjects, in pens in 29, housing was switched during the experiment in 4, and housing type was not specified in 11 BioProjects. The age of sampled birds ranged from 0 to 371 d. In 47 BioProjects, the V3-V4 hypervariable region was sequenced and in 17 BioProjects the V4 hypervariable region. Each of the other hypervariable regions or their combinations was sequenced in less than 5 BioProjects. For 2 BioProjects, the sequenced region was not specified.

**Table 1:** Number of data sets, samples, and microbial features included in the secondary analysis.

Segment	Data sets	Samples	Phyla	Classes	Genera	Functional orthologs
Duodenum	5	103	26	58	543	7,313
Jejunum	18	237	51	143	1,313	8,011
Ileum	24	960	68	190	2,411	8,490
Ceca	63	1,634	37	106	932	7,825
Feces	9	474	58	137	916	7,855

### Taxonomy

**Fig. 1:**
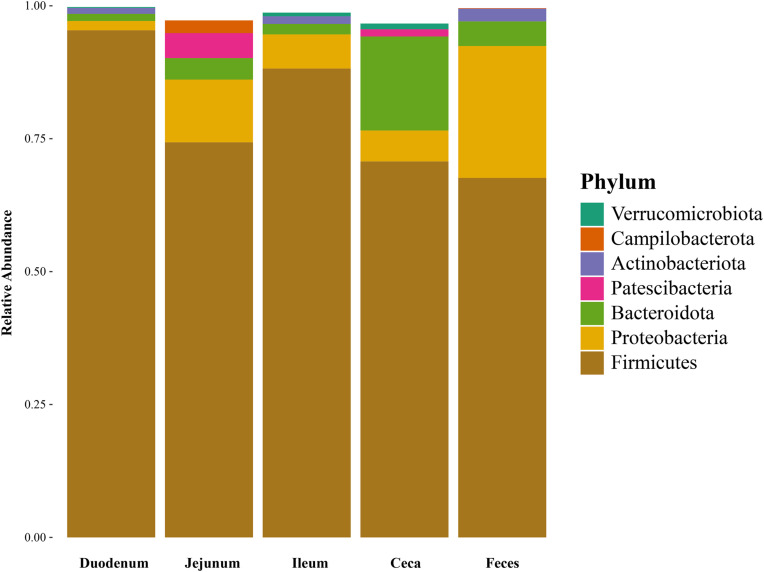
Relative abundance (%) of microbial phyla across intestinal segments averaged at the dataset level. Sample size numbers are shown in Table 1. Each bar represents an intestinal segment, with distinct colors indicating different phyla.

A total of 2,603 unique bacterial genera in 73 unique phyla were detected. Most phyla and genera were detected in the ileum, and the fewest in duodenum (Table 1). In all segments, Firmicutes was the most abundant phylum, followed by Proteobacteria, except in the ceca, where Bacteroidota were the second most abundant (Fig. 1). The relative abundance of other phyla was below 0.05 in all segments.

**Fig. 2:**
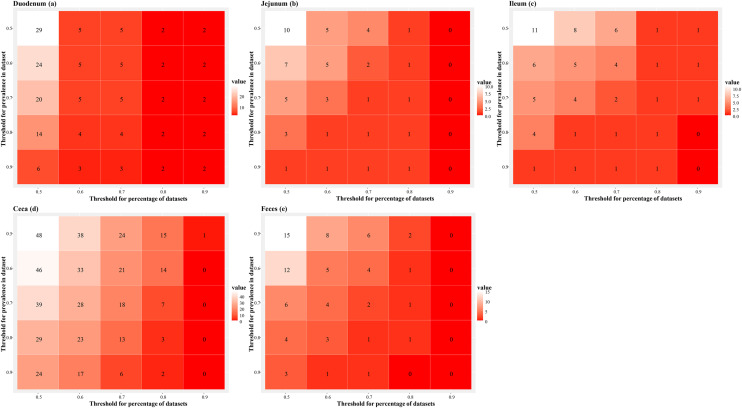
Number of core genera in the duodenum (a), jejunum (b), ileum (c), ceca (d), and feces (e). Core genera were identified based on two thresholds: Threshold 1, the minimum prevalence a genus had to reach within each data set is shown on the y-axis, and threshold 2, the percentage of data sets in which a genus exceeded threshold 1 is shown on the x-axis. Threshold 1 was set at 80% and threshold 2 at 50%.

Two thresholds were used to define core genera. Threshold 1 was the minimum prevalence a genus had to reach within each data set. Threshold 2 was the minimum percentage of data sets in which a genus had to exceed the prevalence set for threshold 1. Increasing threshold 2 decreased the number of core genera in the duodenum (Fig. 2a), jejunum (Fig. 2b), ileum (Fig. 2c), ceca (Fig. 2d), and feces (Fig. 2e) more than increasing threshold 2. Based on these results, threshold 1 was arbitrarily set at 80% and threshold 2 at 50% for subsequent presentation of the results.

The number of core genera was highest in the ceca (Fig. 2d), followed by the duodenum (Fig. 2a) with 29 and 14, respectively. There were less than 5 core genera in the jejunum (Fig. 2b), ileum (Fig. 2c), and feces (Fig. 2e). The percentages of the core genera among all genera detected in the intestinal segment followed the same pattern with values of more than 2% in ceca and duodenum and less than 0.5% in the other segments (Table 2). In contrast, cumulative average relative abundances of the core genera were relatively similar for all segments ranging between 0.46 in the jejunum and 0.82 in the duodenum. Core genera that were detected in more than one intestinal segment included *Lactobacillus* and *Escherichia-Shigella* in all segments, *Ruminococcus torques* group in duodenum, jejunum, ceca and feces, *Eisenbergiella, Lachnoclostridium, Eubacterium coprostanoligenes* group, *Butyricicoccus, Colidextribacter, Erysipelatoclostridium, Ruminococcaceae incertae sedis*, and *Sellimonas* in duodenum and ceca, and *Enterococcus* in ileum and feces. The complete lists of core genera at the selected thresholds are shown in Multimedia Component 4.

**Table 2:** Number of core genera, cumulative percentage of core genera among all genera detected, and cumulative average relative abundance of the core genera in each intestinal segment. A genus was considered a core genus if it was present in more than 80% of samples (threshold 1) and in more than 50% of data sets (threshold 2) of an intestinal segment.

Segment	Number of core genera	Cumulative percentage of genera	Cumulative relative abundance
Duodenum	14	2.35	0.82
Jejunum	3	0.20	0.46
Ileum	4	0.15	0.63
Ceca	29	2.76	0.60
Feces	4	0.39	0.57

### Alpha diversity

**Fig. 3:**
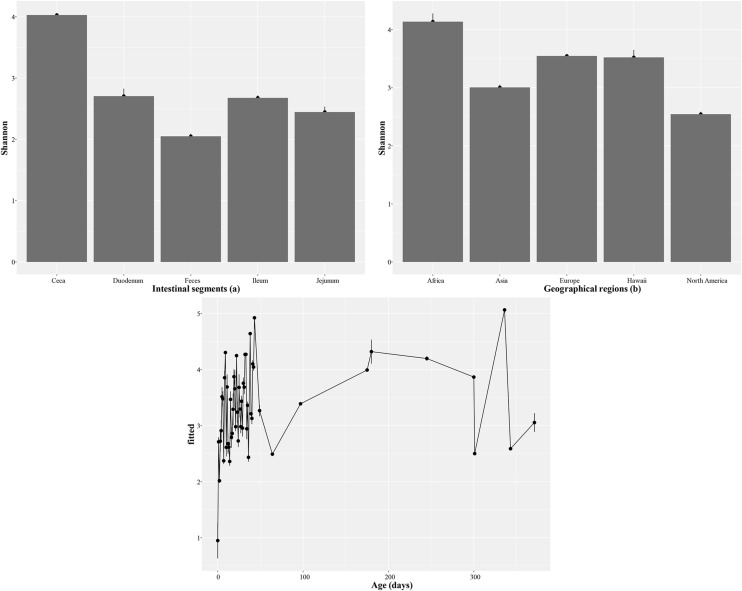
Alpha diversity of the intestinal segments (a), geographical regions (b), and age in days (c) based on the reduced Shannon diversity model.

Linear models were fitted for Shannon diversity and for rank-normalized Simpson’s diversity index (Multimedia Component 5). Adjusted generalized standard error inflation factors (aGSIF) of less than 1.2 indicated that collinearity between predictors was low (Nahhas, 2025). Absolute t-values of less than 2 indicated that bird type, housing, and hypervariable region were not significant for both variables, with hypervariable region V1-V2 being the only exception. Removing bird type, housing, and hypervariable region as predictors resulted in lower AICs which showed a better fit of the models. Alpha diversity was highest in the ceca and lowest in feces (Fig. 3a), as well as highest in Africa and lowest in North America (Fig. 3b). Alpha-diversity increased rapidly during the first week of life, after which no clear trend was observed (Fig. 3c).

### Beta diversity

**Fig. 4:**
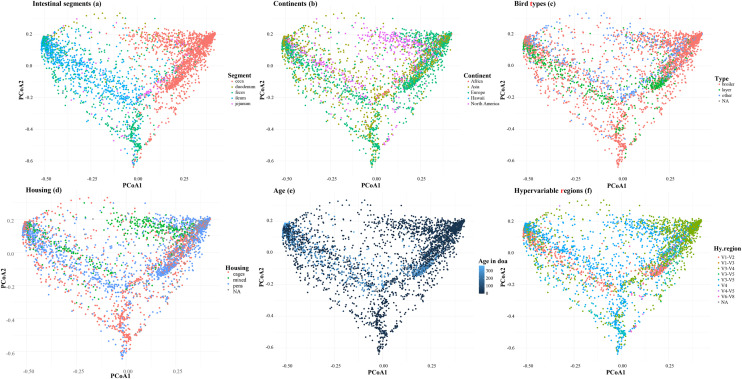
Beta diversity of intestinal segments (a), continents (b), bird types (c), housing (d), age in days, and hypervariable regions (f) as represented by PCoA plots based on Bray-Curtis dissimilarity. Each point represents an individual sample collapsed at the genus level.

The PCoA plot showed only a minor overlap of microbiota from various intestinal segments (Fig. 4a). Groups set apart by different continents (Fig. 4b), bird types (Fig. 4c), housing (Fig. 4d), doa (Fig. 4e), and hypervariable regions (Fig. 4f) were less easily recognized. ANOVA of group dispersions and permutation tests showed highly significant differences in dispersion between groups for all factors except bird type (Supplementary Fig. 2). The unbalanced design violated the assumptions of PERMANOVA (Anderson and Walsh, 2013) so that the beta-diversity patterns have to be regarded as descriptive rather than inferential. For bird type, PERMANOVA results showed a highly significant difference between groups (P < 0.001; Multimedia Component 6).

### Pathways and metabolic prediction

**Fig. 5:**
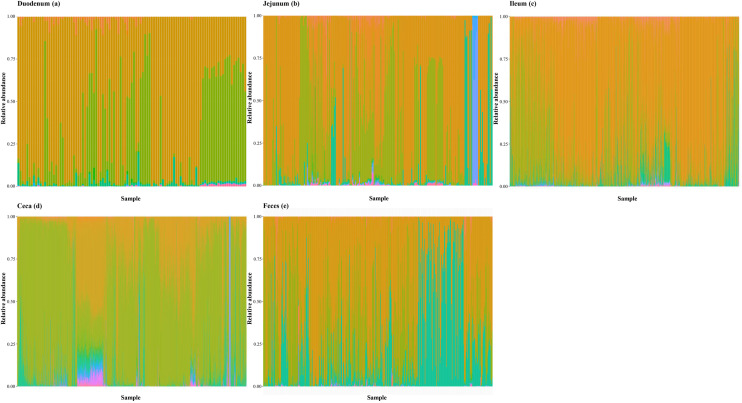
Relative abundance (%) of bacterial classes for each sample in the duodenum (a), jejunum (b), ileum (c), ceca (d), and feces (e). Each column represents a sample, and each color represents a taxon.

**Fig. 6:**
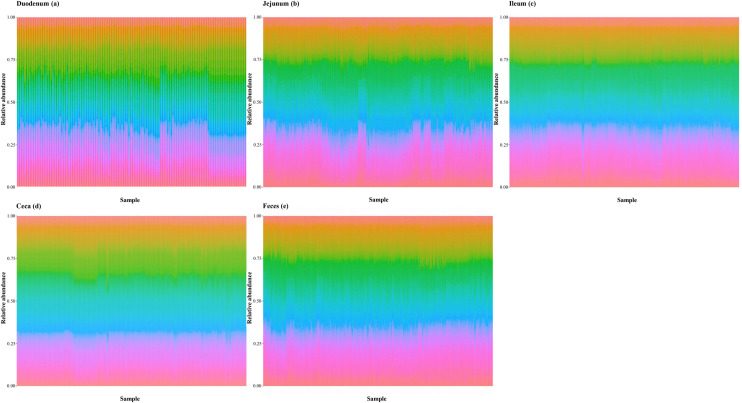
Relative abundance (%) of the most common functional orthologs (FOs) for each sample in the duodenum (a), jejunum (b), ileum (c), ceca (d), and feces (e). Each color represents an FO.

**Fig. 7:**
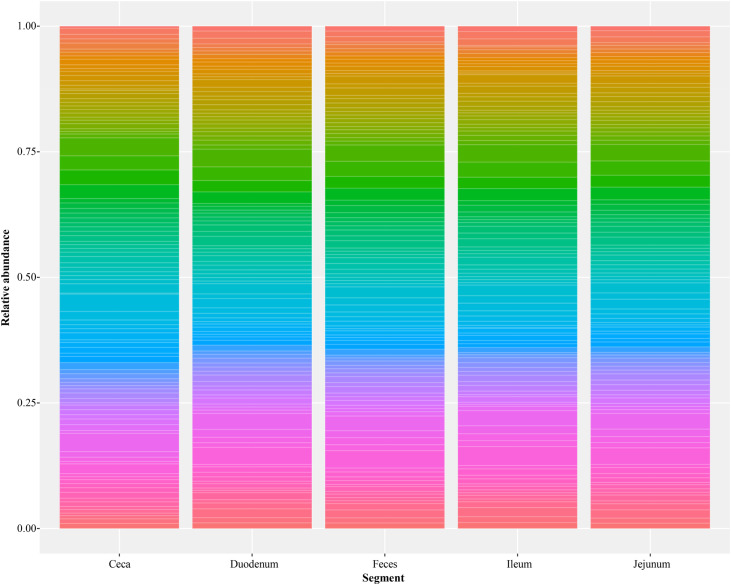
Average relative abundance (%) of the 100 most abundant functional orthologs (FOs) across intestinal segments. Each color represents an FO.

A total of 8,674 unique FOs were detected, with counts ranging from 7,313 in the duodenum to 8,490 in the ileum (Table 1). The relative abundances of bacterial classes (Fig. 5) and FOs (Fig. 6) were plotted for each sample. For each segment, only the most common FOs were plotted, with the number selected matching the number of bacterial classes detected in that segment (Table 1). Class was selected as the taxonomic level as compromise between the very large number of genera and the few phyla. Relative abundances of taxonomic composition showed pronounced differences between samples (Fig. 5), whereas less variation was observed for FOs (Fig. 6). Average relative abundances of the 100 most abundant FOs slightly differed between intestinal segments (Fig. 7). The proportion of nDFOs identified in pairwise comparisons ranged from 29.5% for ileum vs. ceca to 59.1% for duodenum vs. jejunum (Table 3).

**Table 3:** Non-differentially abundant functional orthologs identified by pairwise comparisons of intestinal segments. Values above the diagonal represent the percentages, while values below the diagonal represent absolute counts.

	Duodenum	Jejunum	Ileum	Ceca	Feces
Duodenum		59.1	47.0	47.6	49.4
Jejunum	4,223		32.3	37.3	38.8
Ileum	3,599	2,576		29.5	34.0
Ceca	3,565	2,855	2,413		32.6
Feces	3,701	3,171	2,718	2,712	

The percentage of FOs in the ileum and ceca whose abundances were not significantly influenced by bird type, housing, continent, or doa was determined for all possible pairwise comparisons of these factors. The average percentage of nDFOs when analyzing for the influence of bird type was 37.7% (Table 4), 33.0% for housing (Table 5), 61.1% for continent (Table 6), and 48.0% for doa (Table 7). Considering the individual pairwise comparisons, there were few outliers observed. One notable exception was the comparison between North America and Europe in the ceca, which showed only 38.7% nDFOs compared to much higher percentages for the other pairwise comparisons of continents. Percentages of nDFOs were numerically lower in the ileum than in the ceca, although missing pairwise comparisons prevented a complete assessment.

**Table 4:** Percentage of non-differentially abundant functional orthologs in ileum (I; above diagonal) and ceca (C; below diagonal) identified by pairwise comparisons of bird types.

	Broilers	Layers	Others
Broilers		I: 30.9	I: 30.4
Layers	C: 42.7		I: 37.6
Others	C: 44.7	C: 39.7

**Table 5:** Percentage of non-differentially abundant functional orthologs in ileum (I; above diagonal) and ceca (C; below diagonal) identified by pairwise comparisons of bird housing.

	Cages	Floor pens	Mixed
Cages		I: 32.7	I: 41.6
Floor pens	C: 26.5		I: 25.4
Mixed	C: 38.9	C: 32.7

**Table 6:** Percentages of non-differentially abundant functional orthologs in ileum (I; above diagonal) and ceca (C; below diagonal) identified by pairwise comparisons of continents.

	North America	Europe	Asia	Africa	Hawaii
North America		I: 24.6	I: 44.8	I: NA	I: NA
Europe	C: 38.7		I: 35.5	I: NA	I: NA
Asia	C: 66.4	C: 55.5		I: NA	I: NA
Africa	C: 77.4	C: 72.7	C: 68.4		I: NA
Hawaii	C: 69.1	C: 81.4	C: 79.3	C: 79.5

**Table 7:** Percentages of non-differentially abundant functional orthologs in ileum (I: above diagonal) and ceca (C: below diagonal) identified by pairwise comparisons of age groups in days of age.

	0 – 5	5 – 19	20 – 49	50 or older
0 – 5		I: NA	I: NA	I: 43.8
5 – 19	C: NA		I: NA	I: NA
20 – 49	C: 50.1	C: 47.4		I: NA
50 or older	C: 50.7	C: NA	C: NA	

**Fig. 8:**
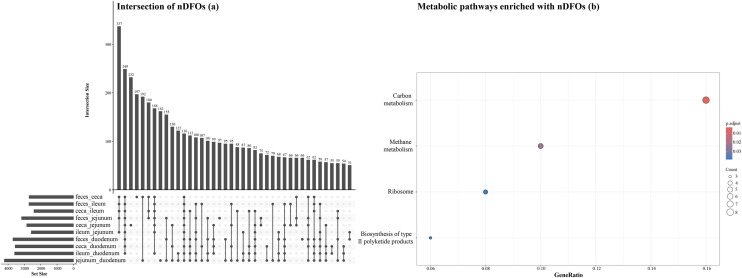
Intersections of non-differentially abundant functional orthologs (nDFOs) detected in pairwise comparisons between intestinal segments (a), and metabolic pathways enriched with nDFOs (b). Gene ratio was defined as genes of interest in the gene set / total genes of interest. p-values for each pathway were adjusted for multiple comparisons using the Benjamini–Hochberg method.

**Fig. 9:**
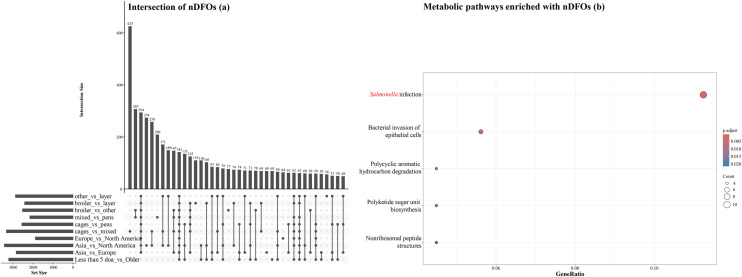
Intersections of non-differentially abundant functional orthologs (nDFOs) detected in all possible pairwise comparisons in the ileum (a), and metabolic pathways enriched with nDFOs (b). Gene ratio was defined as genes of interest in the gene set / total genes of interest. p-values for each pathway were adjusted for multiple comparisons using the Benjamini–Hochberg method.

**Fig. 10:**
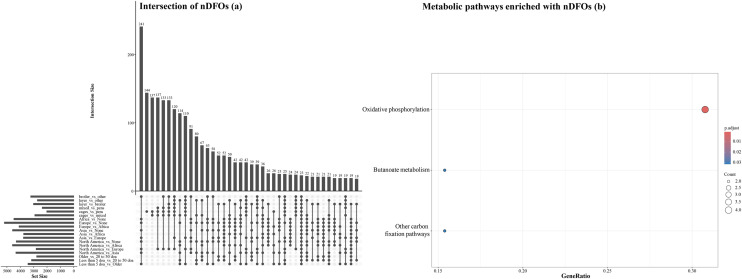
Intersections of non-differentially abundant functional orthologs (nDFOs) detected in all possible pairwise comparisons in the ceca (a), and metabolic pathways enriched with nDFOs (b). Gene ratio was defined as genes of interest in the gene set / total genes of interest. p-values for each pathway were adjusted for multiple comparisons using the Benjamini–Hochberg method.

Intersections of nDFOs that were detected in pairwise comparisons and metabolic pathways of intestinal segments and all possible pairwise comparisons in the ileum and ceca are shown in Fig. 8, Fig. 9, Fig. 10. One hundred and sixteen FOs were identified as not-differentially present in all 10 pair-wise comparisons of intestinal segments (Fig. 8a). When these 116 nDFOs were analyzed using clusterProfiler, 4 metabolic pathways, namely carbon and methane metabolism, ribosome, and biosynthesis of type II polyketide products contained significantly more nDFOs (Fig. 8b). In the ileum, 142 nDFOs were identified in all 10 pairwise comparisons (Fig. 9a) and 5 metabolic pathways were identified including *Salmonella* infection, bacterial invasion of epithelial cells, polycyclic aromatic hydrocarbon degradation, polyketide sugar unit biosynthesis, and nonribosomal peptide structures (Fig. 9b). In the ceca, 23 nDFOs were identified in all 19 pairwise comparisons (Fig. 10a) and 3 pathways were detected which included oxidative phosphorylation, butanoate metabolism, and other carbon fixation pathways (Fig. 10b).

## Discussion

In the last 10 years, investigating the response of the intestinal microbiota to a plethora of influencing factors has arguably been one of the most popular research topics in poultry science. A perceived 100% of studies found that, indeed, the factor under investigation including diet, age, bird type, housing did influence the intestinal microbiota. While this has yielded fascinating insights, interpretation of the results has been difficult and is usually based on taxonomic composition, alpha- and beta-diversity and predicted metabolic function. Differences in taxonomic composition, frequently discussed by picking two or three differentially abundant taxa and referencing studies what these bacteria might do, can rarely be generalized since few bacteria are consistently present in the chicken intestinal tract. Differences in alpha diversity are usually interpreted that higher diversity is indicative of better enteric health, based more on philosophical considerations than on experimental results. In fact, infections with coccidia or *C. perfringens*, the most important intestinal pathogens, do not change alpha diversity (Pietruska et al., 2023). In other instances, infection with pathogens increased alpha diversity (Mudiyanselage et al., 2025). For intestinal microbiota research to remain meaningful, these limitations must be addressed, enabling comparisons across studies and the classification of microbiota as "healthy" or "dysbiotic".

Based on these considerations, the purpose of the present secondary analysis was twofold. First, it was to explore if it might be possible to find markers for a baseline reference, healthy intestinal microbiota, which could serve as a reference for future research and industry practices. Second, it was to assess to what extent age, bird type, housing, geographical location, and methodological differences influence the results and might need to be taken into consideration when interpreting results. To this end, data from 79 BioProjects encompassing 3,562 samples were synthesized, integrating studies across diverse geographic regions, bird types, housing conditions, and sequencing protocols. The presence of certain taxa, alpha diversity in a certain range, clustering in PCoA, as well as the function of the microbiota were considered as parameters to determine a “normal”, healthy intestinal microbial environment.

This approach identified 2,603 unique bacterial genera across 73 unique phyla, showing a much higher diversity than earlier studies. Using 16S rRNA gene sequences, Wei et al. (2013) reported 117 genera and 12 phyla in 3,184 samples from chicken gastrointestinal origin. Recent work by Shen et al. (2024) used metagenomics and confirmed a larger diversity with 1,698 bacterial genera identified from 34 chicken intestinal samples. However, the main phyla were similar. Wei et al. (2013) and Waite and Tayor (2014) reported that Firmicutes were the most abundant phylum in all intestinal segments. These findings align with recent work describing the global chicken gastrointestinal microbiome, which reported dominance by Firmicutes and Bacteroidota and functional conservation across intestinal regions (Burrows et al., 2025). In accordance with that, Firmicutes were the most abundant phylum, followed by Proteobacteria, except in the ceca, where Bacteroidota were more prevalent. This confirms the typical distribution pattern seen in chickens, where Firmicutes play a major role across the gut, except in the ceca (Xiao et al., 2017).

Based on the core microbiota, which are the bacteria found consistently across samples, the ceca had the most core genera (n = 29), followed by the duodenum (n = 14), while fewer than 5 were found in the other intestinal segments. These findings support previous studies suggesting that the ceca host a more stable and diverse community (Zou et al., 2018; Chica Cardenas et al., 2021). This pattern aligns with the core satellite species hypothesis (Gibson et al., 1999), which distinguishes stable, dominant taxa from variable, transient ones. Some genera, like *Lactobacillus* and *Escherichia-Shigella*, appeared in all segments, which confirms their role as common intestinal bacteria in poultry (Stanley et al., 2014). *Lactobacillus* spp. can contribute to nutrient metabolism and absorption in lower sections of the gastrointestinal tract (Oakley et al., 2014; Kogut, 2019), while *Escherichia-Shigella* can have negative correlations with growth performance and nutrient digestibility (Rubio et al., 2014), therefore changing differential abundance of these two genera might be highly indicative of a change in the health status of birds. However, the cumulative average relative abundance of the core genera was similar across all intestinal segments, ranging from 0.0046 to 0.0082 of total microbial communities. While functional importance is not necessarily correlated to abundance, intuitively the low abundance of the core genera still makes their biological significance questionable.

Alpha diversity varied significantly across different intestinal segments and geographical factors, which include differences in temperature, diet and other management practices. It was highest in the ceca and lowest in feces, a pattern consistent with previous studies describing the ceca as a more complex microbial environment characterized by anaerobic conditions and enriched in fermentable substrates (Waite and Taylor, 2014; Borda-Molina et al., 2025). Additionally, alpha diversity was higher in samples from Africa and lower in samples from Asia and North America. This difference may be due to factors such as diet, management practices, or environmental conditions, which vary across regions (Stanley et al., 2014). Glendinning et al. (2024) also found that chicken gut microbiota varied between regions, specifically between regions with different altitudes. Since samples from Africa were underrepresented with only 2 BioProjects versus 36 BioProjects for Asia, 25 for Europe, 13 for North America, and 3 for Hawaii, this finding should be interpreted with caution.

Regarding age, alpha diversity increased rapidly during the first week of life, a result that aligns with previous studies showing that the gut microbiome of chicks establishes quickly after hatching (Torok et al., 2011; Kers et al., 2018). After this period, no trend was visible suggesting that the microbial community reaches stability after early colonization (Wei et al., 2013). In addition and in agreement with findings that using different hypervariable regions can influence alpha diversity (Darwish et al., 2021), use of the V1-V2 hypervariable region changed the alpha diversity. It is unclear whether the lack of influence of the other hypervariable regions reflects true robustness of diversity metrics, limited statistical power for less common regions, or cancellation of region-specific biases at the genus level after aggregation across datasets.

The analyses also indicated that bird type and housing did not significantly influence alpha diversity, so these factors may be less decisive in microbial diversity than intestinal location or geographic origin.

Beta diversity analysis revealed no clear results as, to varying degrees, there was an overlap between groups of all factors, and PERMANOVA was not possible due to differences in dispersion between groups.

The functional pathway analysis detected 8,674 FOs, with numbers ranging from 7,313 in the duodenum to 8,490 in the ileum. The relatively low variation in FO numbers combined with the visually very similar relative abundances of the most abundant FOs was surprising considering the differences in microbial community complexity along the intestinal tract (Waite and Taylor, 2014). While taxonomic composition varied, FO abundances were more consistent, indicating possible functional redundancy, where different taxa perform similar metabolic roles (Tian et al., 2020).

The analysis identified between 29.5% (ileum vs. ceca) and 59.1% (duodenum vs. jejunum) as FOs that were not significantly different between intestinal segments; however, only 116 FOs were identified as not-differentially expressed across all pairwise comparisons indicating gradual transitions. These conserved FOs were mainly involved in 4 metabolic pathways: carbon metabolism, methane metabolism, ribosome biosynthesis, and type II polyketide product biosynthesis. The stability of these core metabolic processes, even as taxonomic composition varied, supports findings by Ballou et al. (2016) demonstrating conserved metabolic networks in poultry microbiomes. However, Pietruska et al. (2023) demonstrated alterations in microbial metabolic pathways in chickens challenged with *C. perfringens*, indicating that intestinal pathogens can influence both community composition and functional potential. Similarly, Ncho (2025) reported that heat stress alters microbial diversity and metabolic pathways, including amino acid metabolism and antioxidant biosynthesis, highlighting that environmental stressors can reshape microbial function.

Examination of external influences revealed that 37.7% of FOs showed no differential expression across bird types, 33.0% were unaffected by housing conditions, 65.8% remained stable regardless of geographic origin, and 48.0% were consistent across age groups. A lower percentage of conserved FOs (38.7%) was observed in the ceca when comparing North America and Europe, possibly due to regional differences in management or diet (Glendinning et al., 2024). In the ileum, 142 conserved FOs included pathways related to *Salmonella* infection and bacterial invasion of epithelial cells. In the ceca, 23 stable FOs were associated with oxidative phosphorylation and butanoate metabolism. These findings are plausible as they align with known segment-specific functions, where the ileum serves as an important immunological interface while the ceca specialize in fermentative metabolism (Oakley et al., 2014; Clavijo and Flórez, 2018).

This secondary analysis has several limitations. First, it is not possible to confirm that all samples originated from truly healthy birds as intended. Although samples from experimentally challenged birds or those receiving atypical diets were excluded, subclinical infections or other stressors may nevertheless have been present. Second, several factors that can vary substantially between flocks without causing disease like lighting, ambient temperature, and diet were not considered. These variables were excluded either because relevant information was unavailable or, in the case of diet, because the complexity of dietary formulations would have made a meaningful analysis impractical. Moreover, these factors are likely correlated with geographic location. Third, the analysis was limited to predicted metabolic functions inferred from 16S rRNA gene sequencing, which is inherently less reliable than direct functional profiling using metagenomics or metabolomics.

In conclusion, this secondary analysis identified some bacterial genera that, with a loose definition, can be considered core genera for each intestinal segment. It also identified FOs and metabolic pathways whose relative abundance was not significantly changed by age, bird type, housing, and geography, supporting the presence of a stable core microbiota under standard production conditions. It is unclear if these genera and FOs can be used to assess a healthy microbial environment in the intestine. Future research will need to take into account more differences in management like lightning schemes, temperature or diet. Future research also needs to show if changes in their relative abundance are correlated to disease and, if so, are caused by disease or conversely if birds with different relative abundances are more susceptible to disease. However, even without this second step, changes in the relative abundance of these genera and FOs detected trials may be more consequential than changes in more variable genera and FOs.

## Funding

This research was funded by United States Department of Agriculture—Agricultural Research Service (USDA-ARS), project number 58-6040-3-016. The authors acknowledge the Alabama Supercomputer Authority for providing high-performance computing resources and technical support, which enabled the data analysis.

## CRediT authorship contribution statement

**Matheus Barros:** Writing – original draft, Methodology, Data curation. **Andrea Pietruska:** Writing – review & editing, Methodology. **Zubair Khalid:** Writing – review & editing, Methodology. **Joseph P. Gulizia:** Writing – review & editing. **Ruediger Hauck:** Writing – review & editing, Project administration, Methodology, Funding acquisition.

## Disclosures

The authors declare that they have no known competing financial interests or personal relationships that could have appeared to influence the work reported in this paper.

## Appendix Supplementary materials

Image, application 1

Image, application 2

Image, application 3

Image, application 4

Image, application 5

Image, application 6

## Footnotes

Supplementary material associated with this article can be found, in the online version, at doi:10.1016/j.psj.2026.106915.
