# Supplementary material for: Secondary analysis of the intestinal microbiota of healthy chickens
Source: Poult Sci. 2026 Apr 13;105(7):106915. doi: 10.1016/j.psj.2026.106915 (PMC13125170; doi:10.1016/j.psj.2026.106915)
Supplement: Supplementary file 1 [file mmc1.docx]

**Identification of studies via databases and registers**

Records removed *before screening*:

Not linked to SRA reads (n = 151)

Records identified from*:

NCBI BioProjects (n = 640)

(file FirstMetaAnalysis 9-5.xlsx)

**Identification**

Records screened

(n = 489)(file Copy of MetaAnalysis-SRAonly (002).xlsx)

Records excluded

(n = 133) (filtered not chicken, not raw sequence, no gut in this file)

Records sought for retrieval

(n = 356)

Publication not retrieved

(n = 80) (filtered no publication found in the file)

**Screening**

Records assessed for eligibility

(n =276) (what remains in this file)

Reports excluded mostly for various reasons, including field studies, no untreated controls, and not 16s rRNA gene sequencing (n = 194) (just calculated the difference)

Studies included

(n = 85) (BioProjects in metadata file)

**Included**

PRJNA689734, PRJNA699633, PRJNA548995, PRJNA701972: I did what maybe we should have done first: I checked the fastq files. You will see that the quality score is weird. They have either all “?” as quality score, which is invalid, or “$”, which would be REALLY bad. We will proceed without this project. (email 02/10/2024); Same thing as previously: load down a fastq file or rather look at one you have and you will see that the data is very questionable – in the sense of the word. Kick ‘em out. (email 02/15/2024)

Sample ERR2486256 in project PRJEB25776 could not be downloaded as a paired end file

Sample ERR4844547 in project PRJEB41302 could not be downloaded as a paired end file

*From:*  Page MJ, McKenzie JE, Bossuyt PM, Boutron I, Hoffmann TC, Mulrow CD, et al. The PRISMA 2020 statement: an updated guideline for reporting systematic reviews. BMJ 2021;372:n71. doi: 10.1136/bmj.n71

For more information, visit: <http://www.prisma-statement.org/>
