# Supplementary material for: Secondary analysis of the intestinal microbiota of healthy chickens
Source: Poult Sci. 2026 Apr 13;105(7):106915. doi: 10.1016/j.psj.2026.106915 (PMC13125170; doi:10.1016/j.psj.2026.106915)
Supplement: Supplementary file 6 [file mmc6.pptx]

## Slide 1
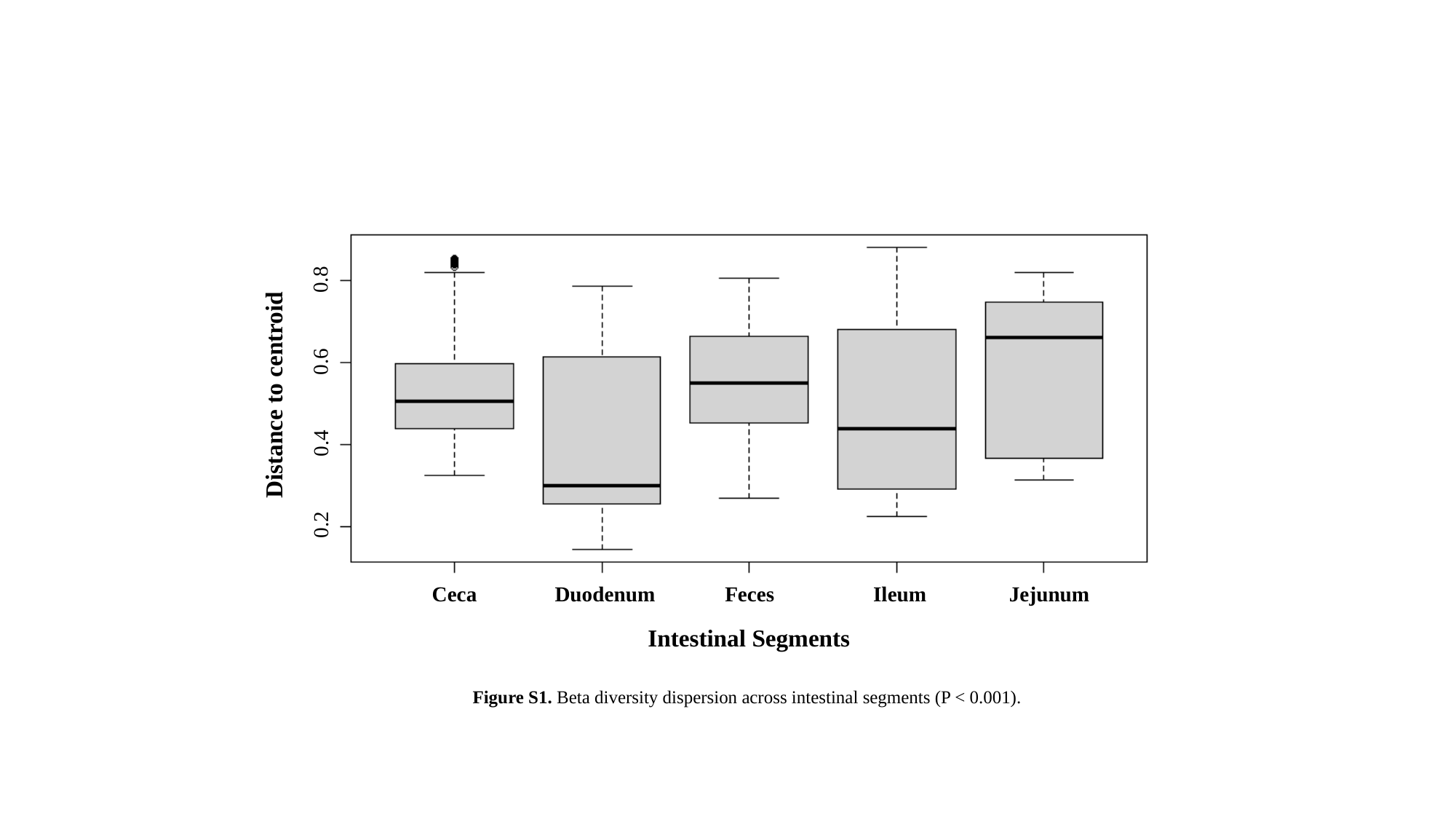

0.8
0.6
Distance to centroid
0.4
0.2
Duodenum
Feces
Ileum
Jejunum
Ceca
Intestinal Segments
Figure S1. Beta diversity dispersion across intestinal segments (P < 0.001).

## Slide 2
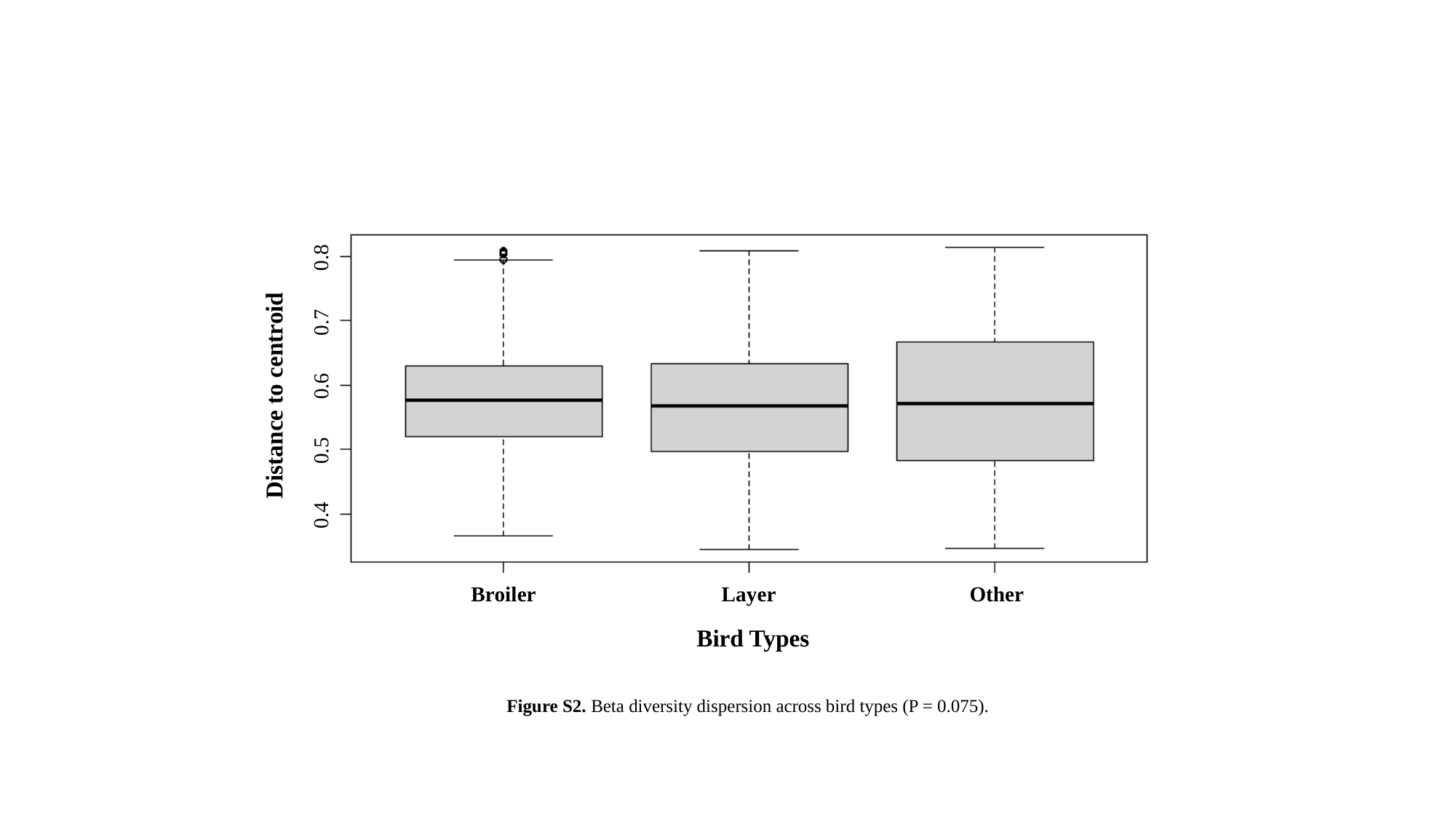

0.8
0.7
0.6
Distance to centroid
0.5
0.4
Broiler
Layer
Other
Bird Types
Figure S2. Beta diversity dispersion across bird types (P = 0.075).

## Slide 3
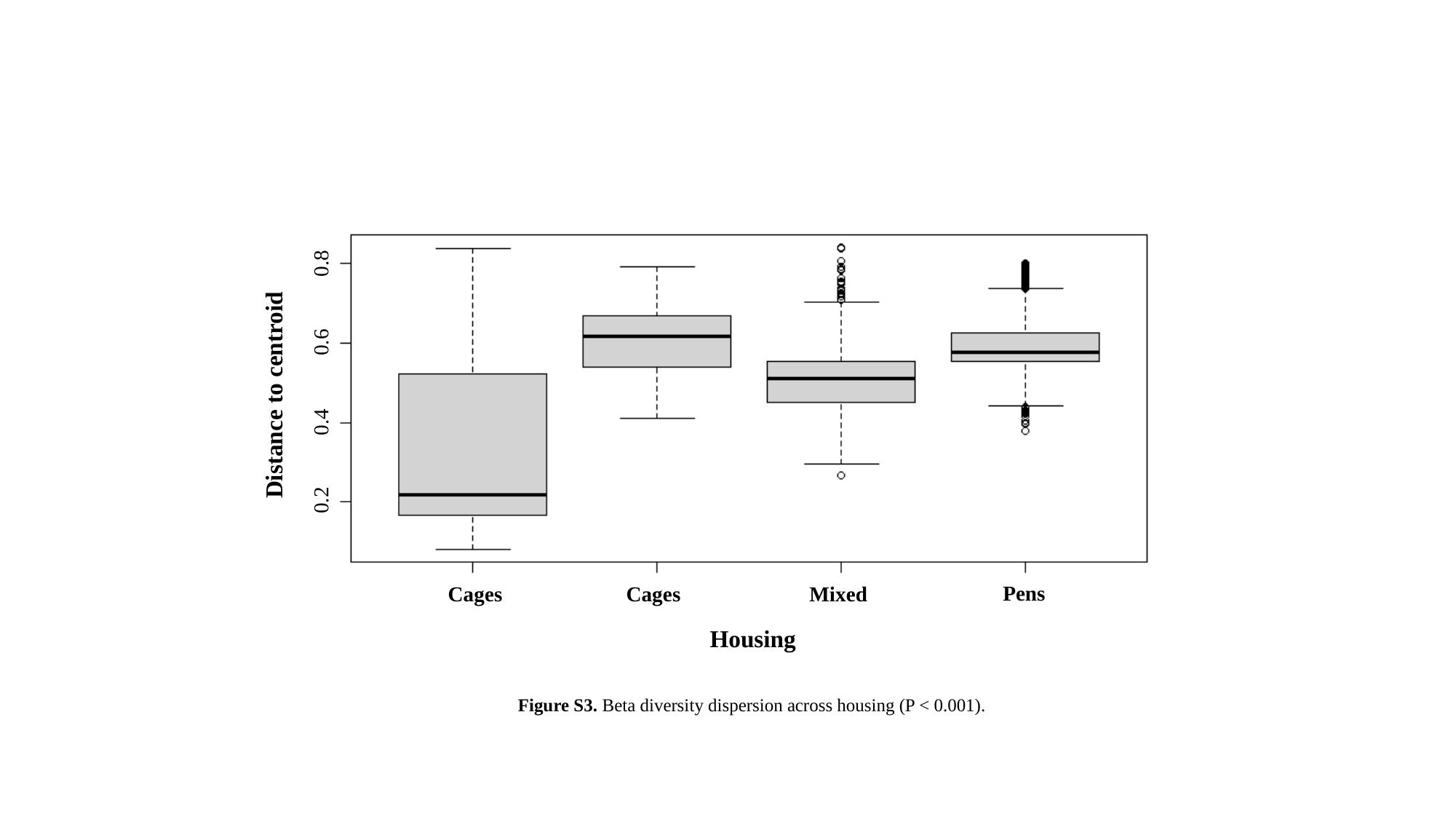

0.8
0.6
Distance to centroid
0.4
0.2
Pens
Mixed
Cages
Cages
Housing
Figure S3. Beta diversity dispersion across housing (P < 0.001).

## Slide 4
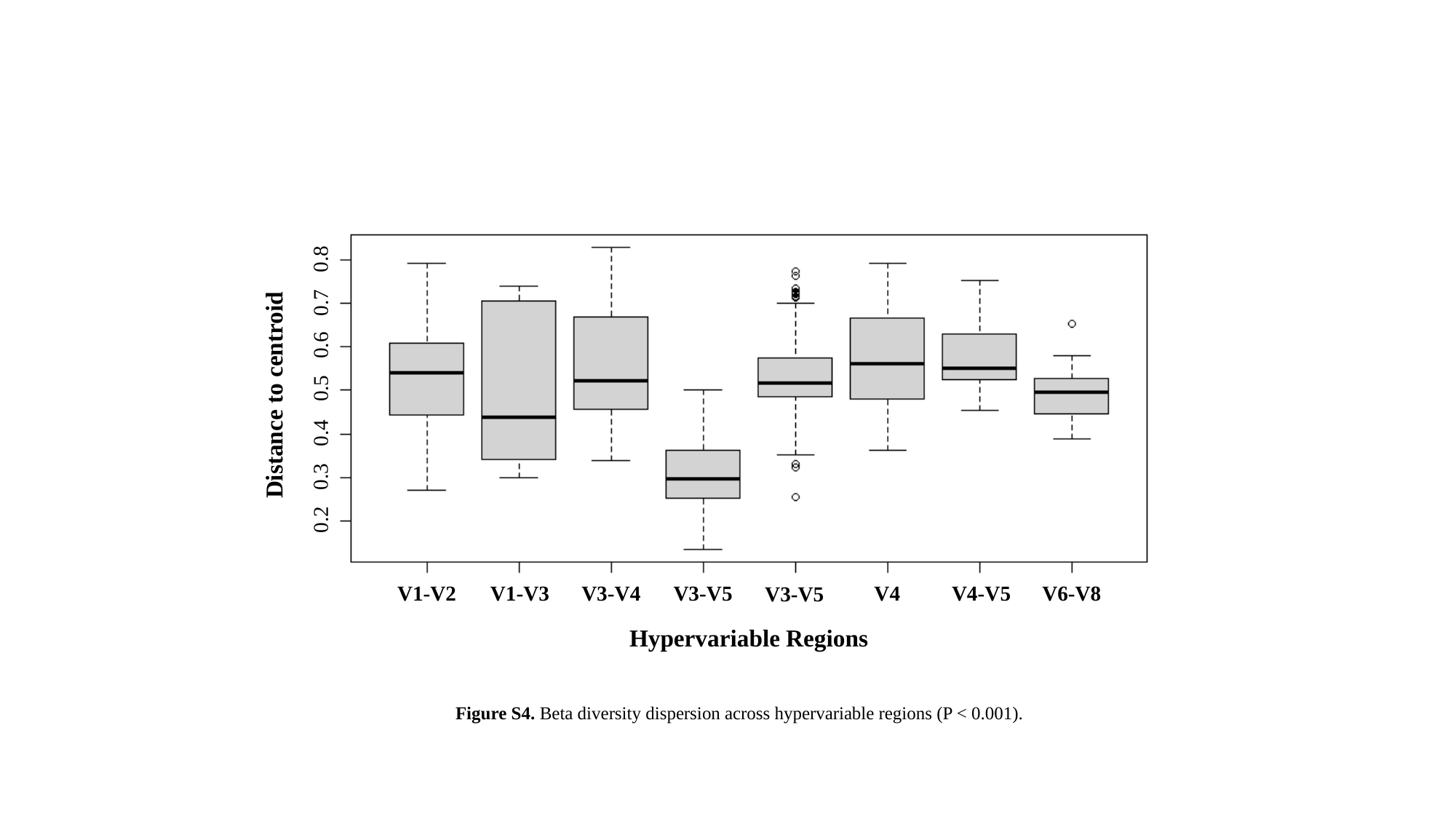

0.8
0.7
0.6
0.5
Distance to centroid
0.4
0.3
0.2
V4-V5
V3-V5
V3-V4
V4
V6-V8
V1-V3
V1-V2
V3-V5
Hypervariable Regions
Figure S4. Beta diversity dispersion across hypervariable regions (P < 0.001).

## Slide 5
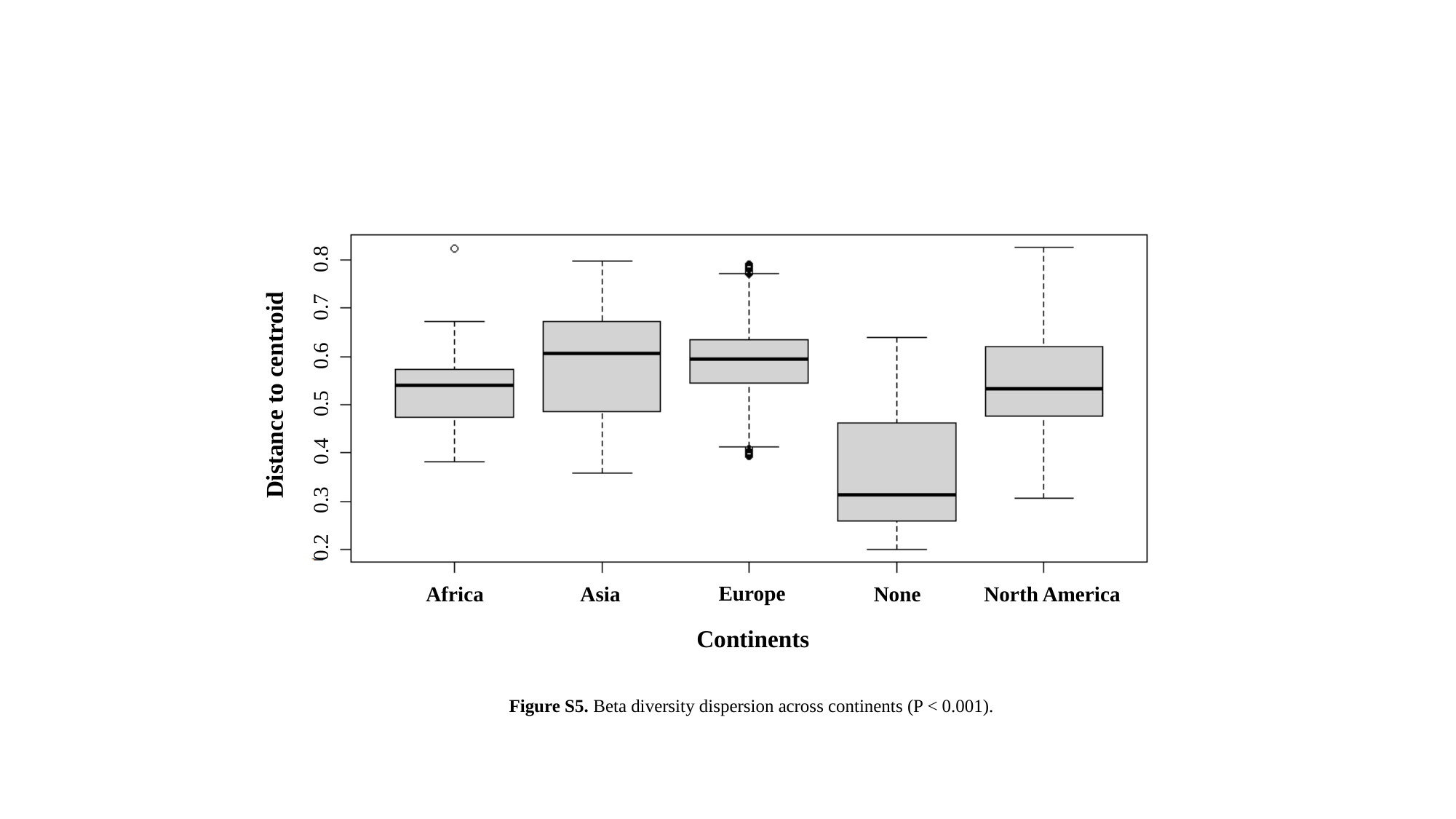

0.8
0.7
0.6
Distance to centroid
0.5
0.4
0.3
0.2
Europe
Africa
Asia
None
North America
Continents
Figure S5. Beta diversity dispersion across continents (P < 0.001).

## Slide 6
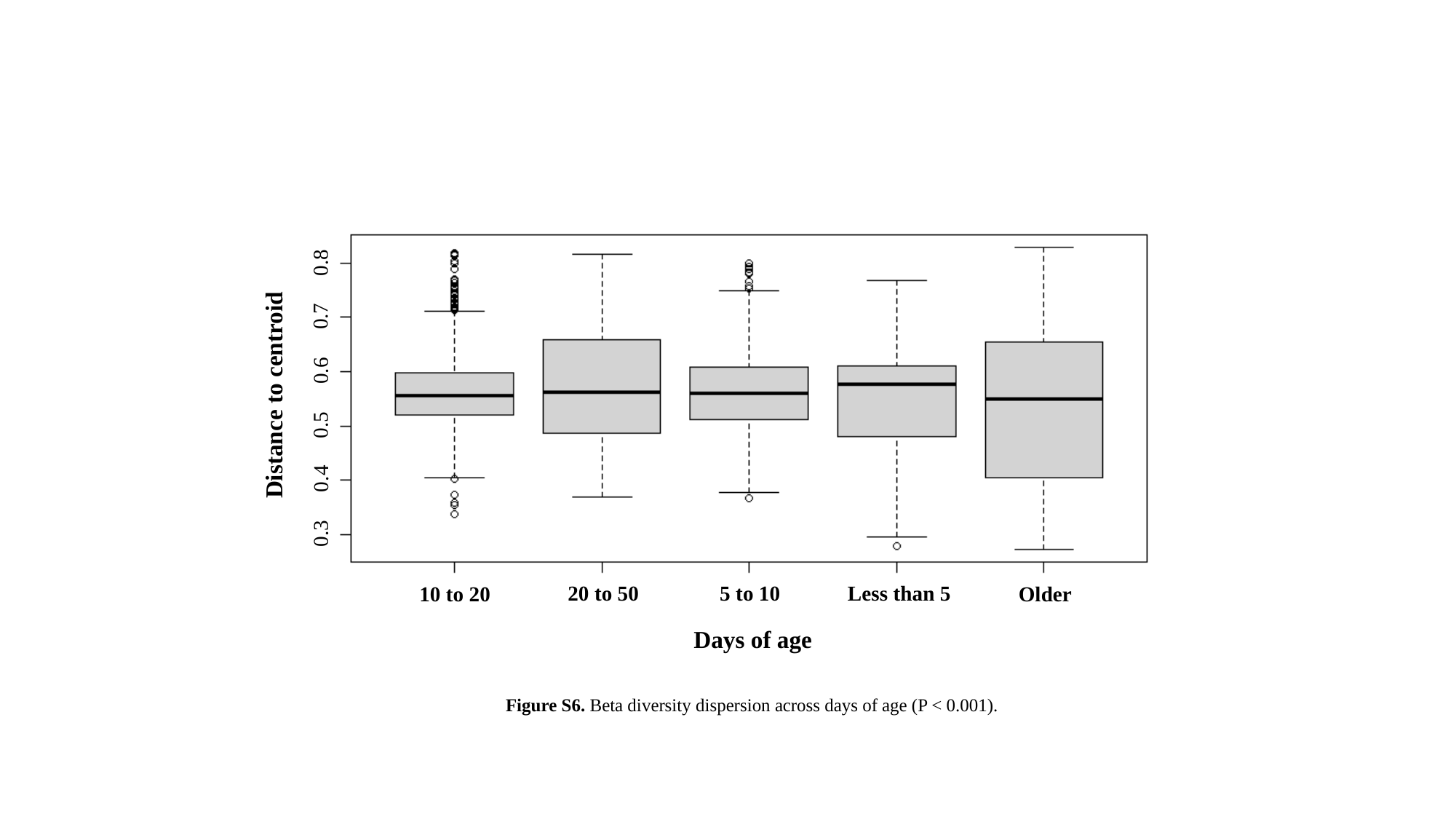

0.8
0.7
0.6
Distance to centroid
0.5
0.4
0.3
5 to 10
Less than 5
20 to 50
Older
10 to 20
Days of age
Figure S6. Beta diversity dispersion across days of age (P < 0.001).

## Slide 7
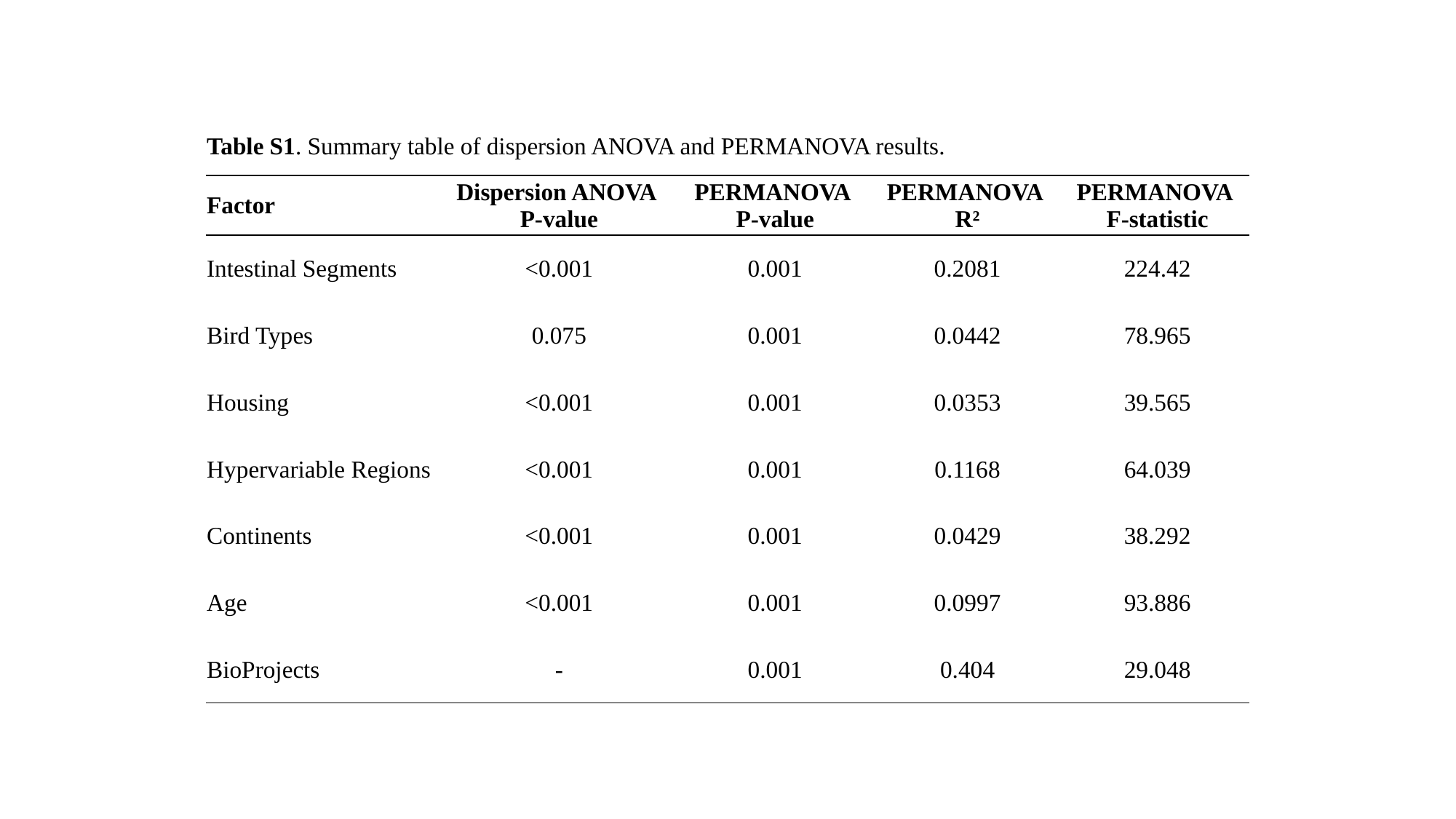

| Table S1. Summary table of dispersion ANOVA and PERMANOVA results. | | | | |
| --- | --- | --- | --- | --- |
| Factor | Dispersion ANOVA P-value | PERMANOVA P-value | PERMANOVA R² | PERMANOVA F-statistic |
| Intestinal Segments | <0.001 | 0.001 | 0.2081 | 224.42 |
| Bird Types | 0.075 | 0.001 | 0.0442 | 78.965 |
| Housing | <0.001 | 0.001 | 0.0353 | 39.565 |
| Hypervariable Regions | <0.001 | 0.001 | 0.1168 | 64.039 |
| Continents | <0.001 | 0.001 | 0.0429 | 38.292 |
| Age | <0.001 | 0.001 | 0.0997 | 93.886 |
| BioProjects | - | 0.001 | 0.404 | 29.048 |
